# Artificial Intelligence-Enabled Electrocardiography Detects Hypoalbuminemia and Identifies the Mechanism of Hepatorenal and Cardiovascular Events

**DOI:** 10.3389/fcvm.2022.895201

**Published:** 2022-06-13

**Authors:** Yung-Tsai Lee, Chin-Sheng Lin, Wen-Hui Fang, Chia-Cheng Lee, Ching-Liang Ho, Chih-Hung Wang, Dung-Jang Tsai, Chin Lin

**Affiliations:** ^1^Division of Cardiovascular Surgery, Cheng Hsin Rehabilitation and Medical Center, Taipei City, Taiwan; ^2^Division of Cardiology, Department of Internal Medicine, Tri-Service General Hospital, National Defense Medical Center, Taipei City, Taiwan; ^3^Department of Family and Community Medicine, Tri-Service General Hospital, National Defense Medical Center, Taipei City, Taiwan; ^4^Department of Internal Medicine, Tri-Service General Hospital, National Defense Medical Center, Taipei City, Taiwan; ^5^Artificial Intelligence of Things Center, Tri-Service General Hospital, National Defense Medical Center, Taipei City, Taiwan; ^6^Medical Informatics Office, Tri-Service General Hospital, National Defense Medical Center, Taipei City, Taiwan; ^7^Division of Colorectal Surgery, Department of Surgery, Tri-Service General Hospital, National Defense Medical Center, Taipei City, Taiwan; ^8^Division of Hematology and Oncology, Tri-Service General Hospital, National Defense Medical Center, Taipei City, Taiwan; ^9^Department of Otolaryngology-Head and Neck Surgery, Tri-Service General Hospital, National Defense Medical Center, Taipei City, Taiwan; ^10^Graduate Institute of Medical Sciences, National Defense Medical Center, Taipei City, Taiwan; ^11^School of Public Health, National Defense Medical Center, Taipei City, Taiwan; ^12^Medical Technology Education Center, School of Medicine, National Defense Medical Center, Taipei City, Taiwan

**Keywords:** artificial intelligence, electrocardiogram, deep learning, hypoalbuminemia, previvor, liver failure events

## Abstract

**Background:**

Albumin, an important component of fluid balance, is associated with kidney, liver, nutritional, and cardiovascular diseases (CVD) and is measured by blood tests. Since fluid balance is associated with electrocardiography (ECG) changes, we established a deep learning model (DLM) to estimate albumin *via* ECG.

**Objective:**

This study aimed to develop a DLM to estimate albumin *via* ECG and explored its contribution to future complications.

**Materials and Methods:**

A DLM was trained for estimating ECG-based albumin (ECG-Alb) using 155,078 ECGs corresponding to albumin from 79,111 patients, and another independent 13,335 patients from an academic medical center and 11,370 patients from a community hospital were used for internal and external validation. The primary analysis focused on distinguishing patients with mild to severe hypoalbuminemia, and the secondary analysis aimed to provide additional prognostic value from ECG-Alb for future complications, which included mortality, new-onset hypoalbuminemia, chronic kidney disease (CKD), new onset hepatitis, CVD mortality, new-onset acute myocardial infarction (AMI), new-onset stroke (STK), new-onset coronary artery disease (CAD), new-onset heart failure (HF), and new-onset atrial fibrillation (Afib).

**Results:**

The AUC to identify hypoalbuminemia was 0.8771 with a sensitivity of 56.0% and a specificity of 90.7% in the internal validation set, and the Pearson correlation coefficient was 0.69 in the continuous analysis. The most important ECG features contributing to ECG-Alb were ordered in terms of heart rate, corrected QT interval, T wave axis, sinus rhythm, P wave axis, etc. The group with severely low ECG-Alb had a higher risk of all-cause mortality [hazard ratio (HR): 2.45, 95% CI: 1.81–3.33] and the other hepatorenal and cardiovascular events in the internal validation set. The external validation set yielded similar results.

**Conclusion:**

Hypoalbuminemia and its complications can be predicted using ECG-Alb as a novel biomarker, which may be a non-invasive tool to warn asymptomatic patients.

## Introduction

Hepatorenal diseases are among the many potential causes of acute kidney injury in patients with acute or chronic liver disease. Patients who develop hepatorenal diseases usually have portal hypertension due to cirrhosis, severe alcoholic hepatitis, or, less often, metastatic tumors (1–3). They also frequently occur in patients with acute liver disease (4), and these patients usually die within a few weeks of the onset of kidney functional impairment if not subjected to immediate intervention (5).

Albumin (Alb) is a protein made by the liver that normally ranges from 3.5 to 5.0 g/dL in adult humans (6) and it represents the hepatorenal status in adult humans. Across disease states, serum Alb concentrations decrease as a result of reduced synthesis and/or increased catabolism such that the protein is considered a negative acute phase reactant (7). Hypoalbuminemia (Alb ≤ 3.5 g/dL) may be a leading biomarker for future hepatorenal diseases in clinical practice (8). Moreover, hypoalbuminemia is also related to nutritional deterioration and disease-related inflammatory responses (9). Previous studies also mentioned the important role of hypoalbuminemia in future cardiovascular events (10). The prevalence of hypoalbuminemia is greater than 70% among elderly hospitalized patients (11), which emphasizes the importance of intensive Alb management.

Although acute hypoalbuminemia may need to be rapidly corrected by albumin and furosemide to manage potential adverse events (12, 13), the treatment of chronic hypoalbuminemia requires focusing on the underlying causes (14). Considering that chronic hypoalbuminemia is common in clinical practice, serum Alb may be an important indicator to remind doctors to notice potential complications. However, serum Alb tests are usually only conducted for suspected malnutrition or hospitalization, which may lead to missing potential cases in patients with subtle symptoms.

Electrocardiogram (ECG) is a non-invasive and convenient way to detect electrical changes at the skin surface when cardiomyocytes depolarize. Hypoalbuminemia may cause edema leading to low voltage on the ECG (15). The ECG findings associated with lower serum Alb levels include abnormal QTc intervals (16). Although these ECG electrical changes may be associated with hypoalbuminemia, it is still difficult to identify hypoalbuminemia by ECG even among experienced physicians.

With developments of deep learning models (DLMs), DLM-enabled ECG is a powerful tool for detecting acute myocardial infarction (17), digoxin toxicity (18), dyskalemias (19), and diabetes mellitus (20). Moreover, a previous study demonstrated that artificial intelligence (AI)-enabled ECG was able to extract predictors of left ventricular dysfunction in patients with a normal ejection fraction (21). AI-enabled ECG also identified a high mortality risk group among patients with normal serum potassium concentrations (22). Moreover, ECG can also be used to predict heart age as a measure of cardiovascular health, and the difference between ECG age and actual age can be used as a biomarker of the risk of death (23). With the help of AI, ECG can not only predict the risk of future disease in healthy people but also enable early preventive interventions to reduce the risk of disease to achieve the purpose of primary prevention for health promotion and special protection.

Therefore the aim of this study was to employ DLM to analyze ECGs for hypoalbuminemia detection. We hypothesized that AI-enabled ECG might also be able to detect hypoalbuminemia if a large annotated database is available, which would help to manage hepatorenal and CVD. Since ECG includes plentiful physical information related to predictors of future cardiovascular diseases, it may also provide additional information on future hepatorenal and CVD. This study aimed to train a DLM to predict Alb using ECG as a novel biomarker called ECG-Alb and to explore its contribution to all-cause mortality, new-onset hypoalbuminemia, new-onset CKD, new-onset hepatitis, CVD mortality, new-onset AMI, new-onset STK, new-onset CAD, new-onset HF, and new-onset Afib in patients with normal serum Alb.

## Materials and Methods

### Data Source and Population

This study was reviewed and approved by the institutional ethics committee of the Tri-Service General Hospital (C202105049). We conducted a retrospective study to develop a DLM and to internally and externally validate its performance. The ECGs from two hospitals, an academic medical center at Neihu District (hospital A) and a community hospital at Zhongzheng District (hospital B), in the Tri-Service General Hospital System, were collected from January 1, 2010 to September 30, 2021. Each ECG was annotated by the nearest Alb concentration ranging from 2.0 to 5.0 g/dL. ECGs without Alb tests within 30 days were excluded from this study.

**FIGURE 1:**
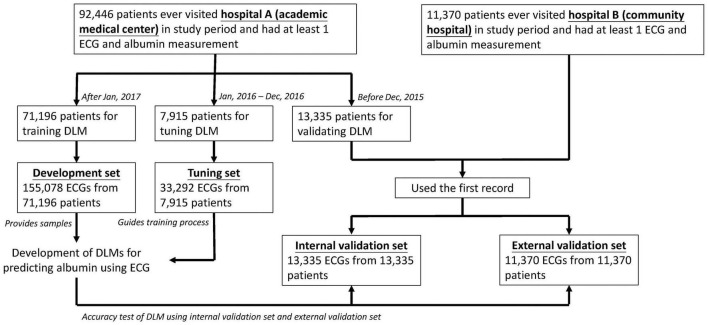
Development, tuning, internal validation, and external validation set generation and ECG labeling of albumin. Schematic of the dataset creation and analysis strategy, which was devised to assure a robust and reliable dataset for training, validating, and testing of the network. Once a patient’s data were placed in one of the datasets, that individual’s data were used only in that set, avoiding “cross-contamination” among the training, validation, and test datasets. The details of the flow chart and how each of the datasets was used are described in “Materials and Methods”.

As shown in Figure 1, we designed the following methods for model development and validation of the DLM. In hospital A, a total of 92,446 patients had at least one ECG and Alb pair in this study. Next, the 71,196 patients who visited hospital A after January 1, 2017 were assigned to the development set, which provided 155,078 ECG records for DLM training. The 7,915 patients from January 1, 2016 to December 31, 2016 were assigned to the training set, which provided 33,292 ECGs for guiding the training process and selecting the critical operating point for diagnosis. Finally, 13,335 patients before December 31, 2015 were assigned to the internal validation set, which only provided ECGs to conduct the accuracy test and follow-up analysis. To verify the extrapolation of the DLM, we also collected 11,370 patients from hospital B with the same inclusion criteria as hospital A for the external validation set.

### Data Collection

In the ECG dataset, we identified patients with at least one standard digital, 500 Hz frequency, 10 s, 12-lead ECG acquired in the supine position during the study period. Alb was measured using the bromocresol green method in the central laboratory. The DLM was trained *via* raw ECG traces. The quantitative measurements and abnormal findings of the ECG were extracted as 31 diagnostic pattern classes and 8 continuous ECG measurements (20). The missing values of the ECG measurement data were imputed using multiple imputations (24). The 31 clinical diagnosis patterns were parsed from the structured findings statements on the basis of the key phrases that are standard within the Philips system. Mild, moderate, and severe hypoalbuminemia were defined as albumin (Alb) of ≤ 3.5, ≤ 3.0, and ≤ 2.5, respectively.

In addition to Alb measurements and 12-lead ECGs, we also collected the relevant blood laboratory values, including glucose (GLU), liver and renal function profiles, c-reactive protein (CRP), complete blood cell count, and lipid profiles. The most recent laboratory test was obtained after enrollment. The disease histories were based on the corresponding International Classification of Diseases, Ninth Revision and Tenth Revision (ICD-9 and ICD-10, respectively) as follows: diabetes mellitus (DM, ICD-9 codes 250.x and ICD-10 codes E08.x to E13.x), hypertension (HTN, ICD-9 codes 401.x to 404.x and ICD-10 codes I10.x to I16.x), hyperlipidemia (HLP, ICD-9 codes 272.x and ICD-10 codes E78.x), chronic kidney disease (CKD, ICD-9 codes 585.x and ICD-10 codes N18.x), acute myocardial infarction (AMI, ICD-9 codes 410.x and ICD-10 codes I21.x), stroke (STK, ICD-9 codes 430.x to 438.x and ICD-10 codes I60.x to I63.x), coronary artery disease (CAD, ICD-9 codes 410.x to 414.x, and 429.2, and ICD-10 codes I20.x to I25.x), heart failure (HF, ICD-9 codes 428.x and ICD-10 codes I50.x), atrial fibrillation (Afib, ICD-9 codes 427.31 and ICD-10 codes I48.x), and chronic obstructive pulmonary disease (COPD, ICD-9 codes 490.x to 496.x and ICD-10 codes J44.9).

The complications of interest in this study were all-cause mortality, new-onset hypoalbuminemia, new-onset CKD, new-onset hepatitis, CVD mortality, new-onset AMI, new-onset STK, new-onset CAD, new-onset HF, and new-onset Afib. For all-cause and CVD mortality, the survival time was calculated with reference to the date of ECG. Patient status (dead/alive) was defined through electronic medical records, which were updated by each hospital activity. Moreover, data for alive visits were censored at the patient’s last known hospital alive encounter to limit bias from incomplete records. A new-onset hypoalbuminemia event was defined as a record of the corresponding ICD codes, with at least 1 record of estimated albumin ≤ 3.5 g/dL. A new-onset CKD event was defined as a record of the corresponding ICD codes, with at least 2 records of estimated glomerular filtration rate (eGFR) ≤ 60 mL/min, or markers of kidney damage (Alb to creatinine ratio ≥ 30 mg/g or positive urine strip test). A new-onset hepatitis event was defined as a record of corresponding ICD codes, at least 1 record of estimated aspartate aminotransferase (AST) or alanine aminotransferase (ALT) > 80 U/L, or Alb Globulin Ratio (A/G) < -1. New-onset CVD events (such as AMI, STK, CAD, HF, Afib) are defined as when the patient was first diagnosed and documented by ICD codes in our hospital electronic medical records. Patients meeting any of the above criteria before the date of the ECG were excluded and defined as having a disease history.

### Deep Learning Model Training

We use the developed ECG12Net, which is an 82-layer convolutional neural network proposed previously (17–19, 25). In this study, we applied the same architecture to train a DLM to estimate ECG-Alb. We used the same training details as in previous studies (17–20). We used the oversampling process based on the inverse prevalence of each Alb interval based on 0.2 g/dL from 2.0 to 5.0 g/dL in the development set.

### Statistical Analysis and Model Performance Assessment

Patient characteristics are presented as the means and standard deviations, numbers of patients, or percentages where appropriate. All statistical analyses were completed in R version 3.4.4. The significance level was set as *p* < 0.05. Scatter plots with mean difference and standard deviation (Diff), Pearson correlation coefficient (r), and mean absolute error (MAE) were used to compare the actual Alb and ECG-Alb. Moreover, the diagnostic value for mild, moderate, and severe hypoalbuminemia was determined in the internal and external validation sets. The area under the ROC curve (AUC), sensitivity (Sens.), specificity (Spec.), positive predictive value (PPV), and negative predictive value (NPV) are presented. The operating point was selected based on the maximum of Yunden’s index in the training set. Due to the different distributions of Alb in the internal and external validation sets, we generated a balanced dataset for each set to ensure the same number of cases for different values of Alb. Moreover, the results of the XGB model are presented, which provided corresponding variable importance rankings to explore the relationship between explainable features and ECG-Alb. Finally, we used multivariable Cox proportional hazard models to analyze the relationship between the baseline characteristics and the outcomes of interest. Hazard ratios (HRs) and 95% confidence intervals (95% CIs) were used for comparisons.

## Results

Table 1 shows the distribution of the basic demographic characteristics, Alb, disease history and laboratory data in the development set, training set, internal validation set and external validation set. Importantly, the internal validation set was significantly younger than the other groups, with a lower proportion of disease history and better laboratory values.

**TABLE 1:** Baseline characteristics.

	Development set	Training set	Internal validation set	External validation set	*P*-value
**Albumin profile**					
Alb (g/dL)	3.6 ± 0.7	3.5 ± 0.6	4.1 ± 0.6	3.6 ± 0.6	<0.001
Alb ≤ 2.5	10,905 (7.0%)	2,469 (7.4%)	278 (2.1%)	612 (5.4%)	<0.001
2.5 < Alb ≤ 3.0	21,657 (14.0%)	5,779 (17.4%)	820 (6.1%)	1,517 (13.3%)	<0.001
3.0 < Alb ≤ 3.5	35,196 (22.7%)	9,698 (29.1%)	1,735 (13.0%)	2,928 (25.8%)	<0.001
3.5 < Alb	87,320 (56.3%)	15,346 (46.1%)	10,502 (78.8%)	6,313 (55.5%)	<0.001
**Demography**					
Sex (male)	90,559 (58.4%)	18,371 (55.2%)	7,535 (56.5%)	6,040 (53.1%)	<0.001
Age (years)	62.7 ± 18.0	69.2 ± 15.9	55.6 ± 18.1	68.2 ± 17.1	<0.001
BMI (kg/m^2^)	24.2 ± 4.3	24.0 ± 4.4	24.3 ± 4.1	24.2 ± 4.3	<0.001
SBP (mmHg)	131.0 ± 27.0	137.0 ± 29.6	130.4 ± 26.2	137.3 ± 28.7	<0.001
DBP (mmHg)	77.5 ± 17.2	76.3 ± 18.9	78.0 ± 16.0	76.3 ± 18.1	<0.001
**Disease history**					
DM	39,627 (25.6%)	13,915 (41.8%)	2,535 (19.0%)	3,891 (34.2%)	<0.001
HTN	55,492 (35.8%)	20,182 (60.6%)	4,189 (31.4%)	6,063 (53.3%)	<0.001
HLP	46,972 (30.3%)	18,040 (54.2%)	2,077 (15.6%)	3,388 (29.8%)	<0.001
CKD	46,401 (29.9%)	15,920 (47.8%)	3,545 (26.6%)	4,807 (42.3%)	<0.001
AMI	8,240 (5.3%)	3,204 (9.6%)	233 (1.7%)	265 (2.3%)	<0.001
STK	23,872 (15.4%)	8,288 (24.9%)	1,434 (10.8%)	2,440 (21.5%)	<0.001
CAD	33,427 (21.6%)	12,832 (38.5%)	2,253 (16.9%)	2,957 (26.0%)	<0.001
HF	18,817 (12.1%)	7,952 (23.9%)	936 (7.0%)	1,424 (12.5%)	<0.001
Afib	9,630 (6.2%)	4,233 (12.7%)	435 (3.3%)	694 (6.1%)	<0.001
COPD	20,085 (13.0%)	8,116 (24.4%)	1,709 (12.8%)	2,781 (24.5%)	<0.001
**Laboratory data**					
GLU (mg/dL)	155.2 ± 97.0	160.5 ± 99.3	129.1 ± 74.3	155.5 ± 99.1	<0.001
HbA1c (%)	6.9 ± 1.9	6.5 ± 1.6	6.1 ± 1.4	6.5 ± 1.6	<0.001
TG (mg/dL)	120.3 ± 83.0	120.6 ± 86.1	120.3 ± 81.5	118.9 ± 85.6	0.309
TC (mg/dL)	159.9 ± 47.7	150.9 ± 45.1	171.8 ± 42.4	156.5 ± 44.5	<0.001
LDL (mg/dL)	97.4 ± 38.1	86.8 ± 36.0	103.6 ± 35.5	92.0 ± 35.9	<0.001
HDL (mg/dL)	44.7 ± 15.7	42.2 ± 14.8	47.8 ± 14.8	44.0 ± 14.8	<0.001
eGFR (mL/min)	72.9 ± 34.8	56.8 ± 35.5	84.3 ± 30.2	68.5 ± 30.6	<0.001
BUN (mg/dL)	25.8 ± 24.7	33.0 ± 28.9	18.8 ± 17.0	23.9 ± 21.2	<0.001
AST (U/L)	43.1 ± 158.4	47.5 ± 173.4	29.1 ± 76.5	38.0 ± 154.5	<0.001
ALT (U/L)	38.3 ± 131.0	36.8 ± 133.5	27.3 ± 51.8	32.7 ± 107.0	<0.001
CRP (mg/L)	6.0 ± 7.8	4.6 ± 6.7	2.4 ± 4.8	4.5 ± 6.8	<0.001
WBC (10^3^/μL)	8.9 ± 7.3	9.4 ± 5.4	7.6 ± 5.1	9.3 ± 6.6	<0.001
PLT (10^3^/μL)	226.9 ± 95.7	215.4 ± 95.4	230.5 ± 76.4	216.2 ± 86.1	<0.001
Hb (mg/dL)	12.3 ± 2.6	11.7 ± 2.6	13.3 ± 2.3	12.5 ± 2.5	<0.001

*Alb, albumin; BMI, body mass index; SBP, systolic blood pressure; DBP, diastolic blood pressure; DM, diabetes mellitus; HTN, hypertension; HLP, hyperlipidemia; CKD, chronic kidney disease; AMI, acute myocardial infarction; STK, stroke, CAD, coronary artery disease; HF, heart failure; AF, atrial fibrillation; COPD, chronic obstructive pulmonary disease; GLU, glucose; HbA1c, glycated hemoglobin; TG, triglyceride; TC, total cholesterol; LDL, low-density lipoprotein cholesterol; HDL, high-density lipoprotein cholesterol; eGFR, estimated glomerular filtration rate; BUN, blood urea nitrogen; AST, aspartate aminotransferase; ALT, alanine aminotransferase; CRP, C-reactive protein; WBC, white blood cell count; PLT, platelet; Hb, hemoglobin.*

**FIGURE 2:**
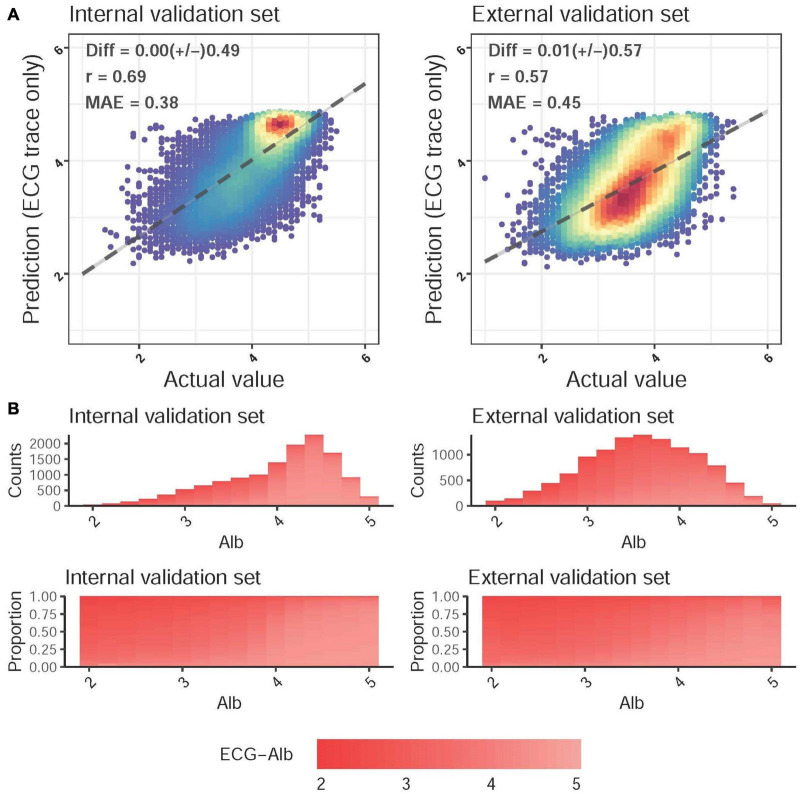
Predicted albumin (ECG-Alb) and actual Alb. **(A)** Scatter plots of ECG-Alb compared to the actual Alb. The x-axis indicates the actual lab measured Alb, and the y-axis presents the ECG-Alb. Red points represent the highest density, followed by yellow, green light blue, and dark blue. We presented the mean difference (Diff), Pearson correlation coefficients (COR), and mean absolute errors (MAE) to demonstrate the accuracy of the DLM. The black lines with 95% conference intervals are fitted *via* simple linear regression. **(B)** The distributions of Alb in the internal and external validation sets. The color gradient from white to red demonstrates the ECG-Alb from normal to low. The top panel shows the original distribution of each dataset, and the bottom panel shows the distribution of ECG-Alb for each actual Alb value.

The scatter plot of Lab-Alb vs. ECG-Alb is presented in Figure 2A. The MAE of Lab-Alb and ECG-Alb in the internal validation set was 0.38 with a correlation of 0.69 and a Diff of 0.00 ± 0.49, and the crude accuracy was slightly reduced in the external validation set (MAE = 0.45, *r* = 0.57, and Diff = 0.01 ± 0.57). The top panel of Figure 2B shows the actual distribution of Lab-Alb in the internal and external sets, which shows that most patients in the internal validation set had a Lab-Alb of ≥ 4.0 g/dL, but most patients in the external validation set had a Lab-Alb of 3.0–4.0 g/dL. This implied that there were more subhealthy patients in the external validation set, leading to a lower crude accuracy compared to the internal validation set. To use the balance distribution to visualize the distribution of ECG-Alb for each Lab-Alb value, the proportions in the internal and external validation sets were similar, as shown in the bottom panel.

**FIGURE 3:**
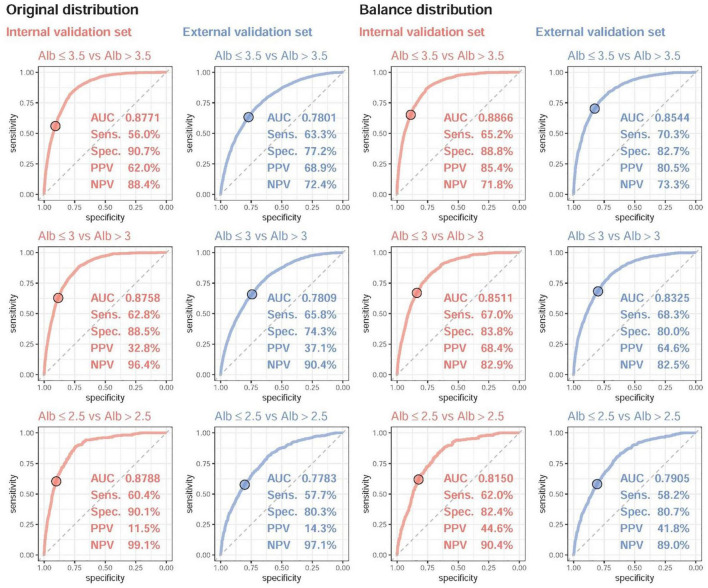
The ROC curve of DLM predictions based on ECG to detect mild to severe hypoalbuminemia. Mild, moderate, and severe hypoalbuminemia were defined as an actual albumin (Alb) of ≤ 3.5, ≤ 3.0, and ≤ 2.5, respectively. The operating point was selected based on the maximum Youden’s index in the tuning set and presented using a circle mark, and the area under the ROC curve (AUC), sensitivity (Sens.), specificity (Spec.), positive predictive value (PPV), and negative predictive value (NPV) were calculated based on it. Due to the different distributions of Alb in the internal and external validation sets, we generated a balanced dataset for each set to ensure the same number of cases for different values of Alb.

Figure 3 shows the accuracy of the DLM for detecting mild, moderate, and severe hypoalbuminemia. The AUC of mild hypoalbuminemia detection was 0.8771, with a Sens of 56.0%, a Spec of 90.7%, a PPV of 62.0%, and an NPV of 88.4%. The AUC values were similar for detecting moderate (0.8758) and severe (0.8788) cases. The crude accuracies in the external validation were much lower than those in the internal validation set (mild hypoalbuminemia, AUC = 0.78, Sens = 63.3%, Spec = 68.9%, PPV = 68.9%, NPV = 72.4%), which may be due to more subhealthy patients in the external validation set. We conducted balance analysis for each set based on the distributions presented in Figure 2B, and the accuracies of the internal and external validation sets were similar in detecting mild, moderate, and severe cases, which implied that the difference in crude accuracies might be primarily due to the inconsistent original Lab-Alb distribution. Interestingly, the AUCs were lower in detecting severe cases than in detecting mild cases in both the internal and external validation sets, which may indicate that major changes in ECG were present in patients with a Lab-Alb of ≤ 3.5 g/dL.

**FIGURE 4:**
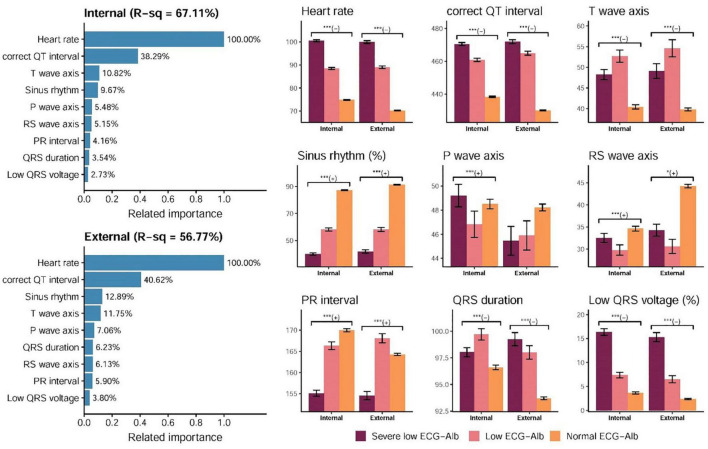
Relationship between the selected ECG features and predicted albumin (ECG-Alb). The related importance is based on the information gain of the XGB model, and the R-square (R-sq) is the coefficient of determination to use selected ECG features for predicting ECG-Alb. The AI-ECG predictions were classified as normal ECG-Alb, low ECG-Alb, and severe low ECG-Alb based on the operating points, as in the previous ROC curve analysis. The analyses are conducted both in internal and external validation sets (*p for trend < 0.05; ^***^p for trend < 0.001).

The details of importance between all ECG features and ECG-Alb based on the information gain of the XGB model are shown in Supplementary Figure 1. The R-squared values were 67.88 and 57.90% based on all traditional ECG features in the internal and external validation sets, respectively. We selected the most important 9 ECG features related to ECG-Alb as shown in Figure 4, and the R-squares were similar compared to the use of all ECG features (67.11%/56.77% in the internal/external validation set). The most important ECG features contributing to ECG-Alb were ordered as heart rate, corrected QT interval, T wave axis, sinus rhythm, P wave axis, etc., in the internal validation set. The heart rate of the severe low group was the highest, followed by the low group and the normal group. The heart rate, corrected QT interval, and low QRS voltage (%) were higher in the severe low ECG-Alb group, but the T wave axis was higher in the low ECG-Alb group.

**FIGURE 5:**
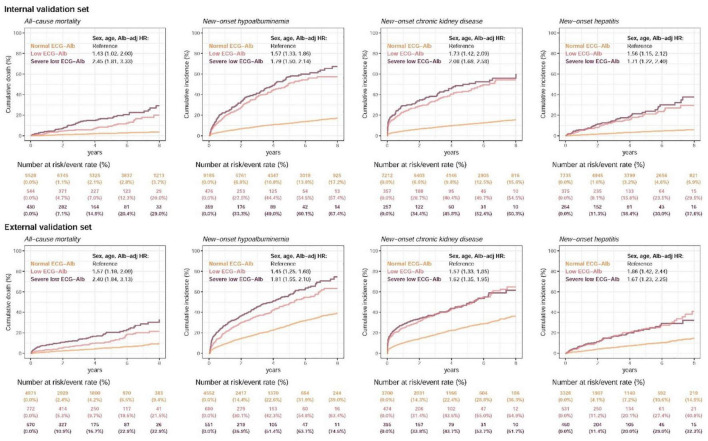
Long-term incidence of developing new-onset malnutrition events in patients with an initially normal albumin (Alb) of > 3.5 g/dL stratified by AI-ECG prediction. The AI-ECG predictions were classified as normal ECG-Alb (yellow line), low ECG-Alb (pink line), and severe low ECG-Alb (burgundy line) based on the operating points, as in the previous ROC curve analysis. The analyses were conducted both in the internal and external validation sets. The table shows the at-risk population and cumulative risk for the given time intervals in each risk stratification.

Figure 5 and Supplementary Figure 2 show the prognostic value of ECG-Alb in patients with an initially normal serum Alb to emphasize the additional prognostic value of ECG-Alb. Table 2 summarized the risk in patients with severe low, low, and normal ECG-Alb on hepatorenal and cardiovascular events. Severe low ECG-Alb group had significant higher risk compared to the normal ECG-Alb group with an HR of 2.45 (95% CI: 1.81–3.33) on all-cause mortality in the internal validation set, and obvious dose-effect relationship was also presented from the HR of the low ECG-Alb group (1.43, 95% CI: 1.02–2.00) to the HR of the severe low ECG-Alb group. This relationship was also validated in the external validation set. Other hepatorenal events and found a similar trend in new-onset hypoalbuminemia, new-onset CKD, and new-onset hepatitis. The cardiovascular events, CVD mortality, new-onset AMI, new-onset STK, new-onset CAD, new-onset HF, and new-onset Afib, were also associated with lower ECG-Alb groups. The additional adjustment considering sex, age, BMI, SBP, SBP, HDL, LDL, DM, Alb also shows the similar trend, which revealed the ability of ECG-Alb to identify hepatorenal and CVD predictors.

**TABLE 2:** Cox proportional hazards model HR and 95% CI Estimates for new-onset hepatorenal and cardiovascular events in different adjustment model.

	Model 1			Model 2		
	Normal ECG-Alb	Low ECG-Alb	Severe low ECG-Alb	Normal ECG-Alb	Low ECG-Alb	Severe low ECG-Alb
**Internal validation set**						
All-cause mortality	Reference	1.43 (1.02, 2.00)	2.45 (1.81, 3.33)	Reference	1.40 (1.00, 1.97)	2.37 (1.74, 3.23)
New-onset hypoalbuminemia	Reference	1.57 (1.33,1.86)	1.79 (1.33, 1.86)	Reference	1.49 (1.26,1.77)	1.65 (1.37, 1.97)
New-onset CKD	Reference	1.73 (1.42, 2.09)	2.08 (1.68, 2.58)	Reference	1.60 (1.32, 1.94)	1.96 (1.59, 2.44)
New-onset hepatitis	Reference	1.56 (1.15, 2.12)	1.71 (1.22, 2.40)	Reference	1.46 (1.07, 1.98)	1.59 (1.13, 2.23)
CVD mortality	Reference	1.93 (0.84, 4.43)	5.17 (2.64, 10.13)	Reference	1.65 (0.71, 3.81)	4.56 (2.31, 8.98)
New-onset AMI	Reference	1.49 (0.89, 2.49)	1.79 (1.04, 3.08)	Reference	1.34 (0.80, 2.25)	1.57 (0.91, 2.71)
New-onset STK	Reference	1.48 (1.11, 1.97)	1.23 (0.88, 1.73)	Reference	1.42 (1.06, 1.89)	1.19 (0.85, 1.68)
New-onset CAD	Reference	1.77 (1.41, 2.22)	1.43 (1.08, 1.90)	Reference	1.72 (1.37, 2.16)	1.37 (1.03, 1.82)
New-onset HF	Reference	1.96 (1.44, 2.68)	3.21 (2.38, 4.34)	Reference	1.87 (1.37, 2.55)	3.06 (2.26, 4.15)
New-onset Afib	Reference	2.69 (1.97, 3.69)	3.17 (2.29, 4.40)	Reference	2.61 (1.90, 3.58)	3.11 (2.24, 4.32)
**External validation set**						
All-cause mortality	Reference	1.57 (1.18, 2.09)	2.40 (1.84, 3.13)	Reference	1.62 (1.22, 2.16)	2.45 (1.87, 3.20)
New-onset hypoalbuminemia	Reference	1.45 (1.25, 1.68)	1.46 (1.26, 1.70)	Reference	1.46 (1.26, 1.70)	1.77 (1.52, 2.06)
New-onset CKD	Reference	1.57 (1.33, 1.85)	1.62 (1.35, 1.95)	Reference	1.54 (1.30, 1.82)	1.60 (1.33, 1.92)
New-onset hepatitis	Reference	1.86 (1.42, 2.44)	1.67 (1.23, 2.25)	Reference	1.86 (1.41, 2.44)	1.61 (1.19, 2.18)
CVD mortality	Reference	2.07 (1.02, 4.22)	3.61 (1.91, 6.83)	Reference	2.11 (1.03, 4.31)	3.75 (1.97, 7.15)
New-onset AMI	Reference	1.47 (0.93, 2.33)	1.50 (0.91, 2.46)	Reference	1.48 (0.94, 2.36)	1.53 (0.93, 2.51)
New-onset STK	Reference	0.83 (0.59, 1.16)	1.47 (1.10, 1.98)	Reference	0.82 (0.59, 1.15)	1.49 (1.11, 1.99)
New-onset CAD	Reference	1.17 (0.92, 1.49)	1.16 (0.90, 1.51)	Reference	1.15 (0.90, 1.47)	1.18 (0.91, 1.47)
New-onset HF	Reference	2.53 (1.95, 3.29)	2.32 (1.74, 3.09)	Reference	2.52 (1.94, 3.28)	2.30 (1.72, 3.07)
New-onset Afib	Reference	2.44 (1.81, 3.31)	2.47 (1.79, 3.40)	Reference	2.40 (1.78, 3.25)	2.48 (1.79, 3.43)

*Model 1: sex, age, Alb adj HR.*

*Model 2: sex, age, BMI, SBP, SBP, HDL, LDL, DM, Alb adj HR.*

## Discussion

The DLM-enabled ECG accurately predicted Lab-Alb to identify mild to severe hypoalbuminemia. Interestingly, the initial external validation showed a significant reduction in AUCs, but the AUCs were revised in balance distribution analysis. Importantly, ECG-Alb provided additional information on clinical outcomes. Patients with lower EGC-Alb had a higher risk of adverse events, such as all-cause mortality, new-onset hypoalbuminemia, new-onset CKD, new-onset hepatitis, CVD mortality, new-onset AMI, new-onset STK, new-onset CAD, new-onset HF, and new-onset Afib. To the best of our knowledge, this is the first study of an AI-ECG system to estimate Lab-Alb.

Blood albumin plays important roles in cardiac function. Previous studies revealed that hypoalbuminemia might lead to edema (26), pulmonary congestion (27), myocardial edema and deterioration of myocardial function (28), diuretic resistance and fluid retention (29), and loss of antioxidant functions and anti-inflammatory functions (30). The heart rate may become elevated when fluid retention occurs, consistent with our findings. Moreover, the QRS amplitude was significantly increased in human studies after albumin infusions. QRS amplitude changes are caused by changes in the serum protein concentration (22). There is also evidence that low serum albumin can lead to changes in QRS amplitudes (31). Hypoalbuminemia was also associated with prolonged QT intervals and T waves with a deceleration accentuated by T waves (16, 32). Therefore, patients with low serum albumin may be detected by the related ECG changes. However, human experts cannot diagnose hypoalbuminemia *via* ECG. AI-enabled ECG provided an opportunity to reveal the relationship between ECG and Lab-Alb, which revealed new medical knowledge of ECG in this study.

The accuracy of AI-ECG was initially significantly reduced in the external analysis, which was often encountered in previous AI-ECG studies. A DLM was trained at the Mayo Clinic to identify subjects with left ventricular dysfunction and initially achieved a sensitivity of 0.86, a specificity of 0.86, and an AUC of 0.93 (21). However, further study showed a lower AUC of 0.82 in Russia (33). The possible reason for this reduction might be due to inconsistent demographic distributions in different populations. A previous study found that gender imbalance led to a decrease in the performance of the model (34). Fortunately, the sex distribution in this study was similar in the internal and external validation sets. However, the population in the external validation set was older than that in the internal validation set, with a higher prevalence of chronic diseases and cardiopulmonary diseases, which may also cause AUC reduction. We thought that the AUC reduction might be due to inconsistent Lab-Alb distributions between the two validation sets. The Lab-Alb was largely concentrated above 4 g/dL in the internal validation set, which resulted in better differentiation of the model in the classification with a cut point of 3.5 g/dL. In contrast, the concentration of Lab-Alb in the external validation set was primarily distributed from 3 to 4 g/dL. Therefore, it was harder to distinguish patients with a Lab-Alb of ≤ 3.5 g/dL in the external validation set. The AUCs in the internal and external analyses were similar after Lab-Alb distribution adjustment. This study revealed a new possibility to explain the performance reduction in external analysis. Future studies should consider investigating disease severity in similar situations.

Serum albumin concentration is an important indicator of mortality risk in many populations, including healthy subjects and patients with acute or chronic illness. The risk of mortality increased from 24 to 56% for each 0.25 g/dL decrement in serum albumin concentration (35). Low albumin levels at admission were associated with increased short- and long-term mortality in hospitalized patients (36). Hypoalbuminemia was associated with increased 30-day all-cause mortality in acutely admitted medical patients (37). Moreover, Serum albumin is also a well-known biomarker of CVD and adverse cardiovascular events (10). ECG-Alb also contributed to predictions of future hepatorenal and cardiovascular events in this study, especially in false-positive predictions among patients with normal Lab-Alb. AI-ECG-positive patients without left ventricular dysfunction had a four-fold higher risk of developing future left ventricular dysfunction than those with negative screens (21). A similar phenomenon was shown in AI-ECG-K^+^ research in which ECG-based hypo- and hyperkalemia provided additional information on all-cause mortality, hospitalizations, and emergency department revisits (22). Moreover, the predicted ECG-age gap from chronological age was also a mortality risk predictor (23), and further study revealed that it was associated with more CVD-related outcomes (38). The consistent findings of this study validated that AI-ECG may identify the extensive CVD predictors mentioned in a previous study (39).

Certain limitations should be mentioned. First, this study was a retrospective study. A prospective study is needed to validate its efficacy in the community. Second, a low-sensitivity ECG-Alb, which is not recommended for risk-scanning procedures. On the other hand, ECG-Alb has a specificity of 90.7%, which is comparable to a positive predictive value of more than 60%. Third, ECG characteristics may vary in different races (40). An international study involving diverse racial and ethnic groups is necessary to validate the accuracy of ECG-Alb. fourth, although ECG-Alb was associated with a higher risk of hepatorenal diseases, it is not a feasible way to treat abnormal ECG-Alb. Finally, our ECG-Alb must become more transparent due to the DLM’s “black box” (41). The correlation and explainability of ECG features with hypoalbuminemia needs to be investigated in future studies.

## Conclusion

In conclusion, our ECG-Alb is a new management tool for hepatorenal diseases, including hypoalbuminemia warning and future hepatorenal and cardiovascular event prediction. It provides a simple, low-cost, and non-invasive method to monitor the serum albumin concentration. Moreover, we provided an opinion to explain the reduction of model accuracy in the external analysis, which may be considered in future DLM studies.

## Data Availability Statement

The original contributions presented in the study are included in the article/Supplementary Material, further inquiries can be directed to the corresponding author/s.

## Ethics Statement

The studies involving human participants were reviewed and approved by the Institutional Ethics Committee of the Tri-Service General Hospital (C202105049). Written informed consent for participation was not required for this study in accordance with the national legislation and the institutional requirements.

## Author Contributions

Y-TL, D-JT, and CL contributed with statistical analysis, interpretation of results, and drafted and revised the manuscript. Y-TL analyzed the data and wrote the first draft. D-JT and CL contributed substantially to revise the subsequent versions and had final responsibility for the decision to submit for publication. C-SL and C-HW provided clinical expertise. W-HF contributed with data processing from clinical perspectives. C-CL, C-LH, and C-HW contributed with contributed with acquisition of data. CL designed the deep learning models, led the development of the manuscript, and conceived the work. All authors have read and agreed to the published version of the manuscript.

## Conflict of Interest

The authors declare that the research was conducted in the absence of any commercial or financial relationships that could be construed as a potential conflict of interest.

## Publisher’s Note

All claims expressed in this article are solely those of the authors and do not necessarily represent those of their affiliated organizations, or those of the publisher, the editors and the reviewers. Any product that may be evaluated in this article, or claim that may be made by its manufacturer, is not guaranteed or endorsed by the publisher.
