# Supplementary figures and images for: Artificial Intelligence-Enabled Electrocardiography Detects Hypoalbuminemia and Identifies the Mechanism of Hepatorenal and Cardiovascular Events
Source: Front Cardiovasc Med. 2022 Jun 13;9:895201. doi: 10.3389/fcvm.2022.895201 (PMC9234125; doi:10.3389/fcvm.2022.895201)

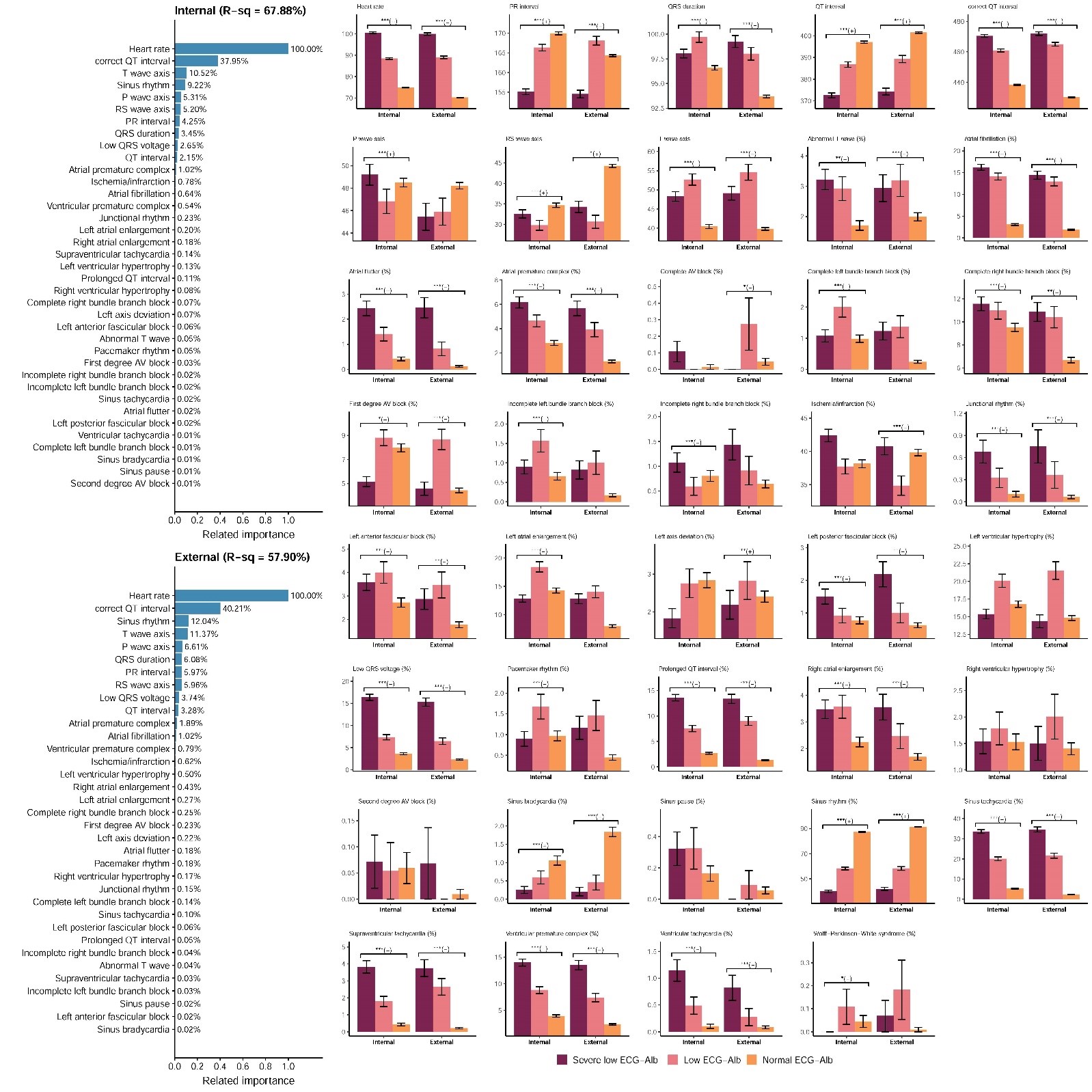

Supplement: Supplementary file 1 [file Image_1.jpg]

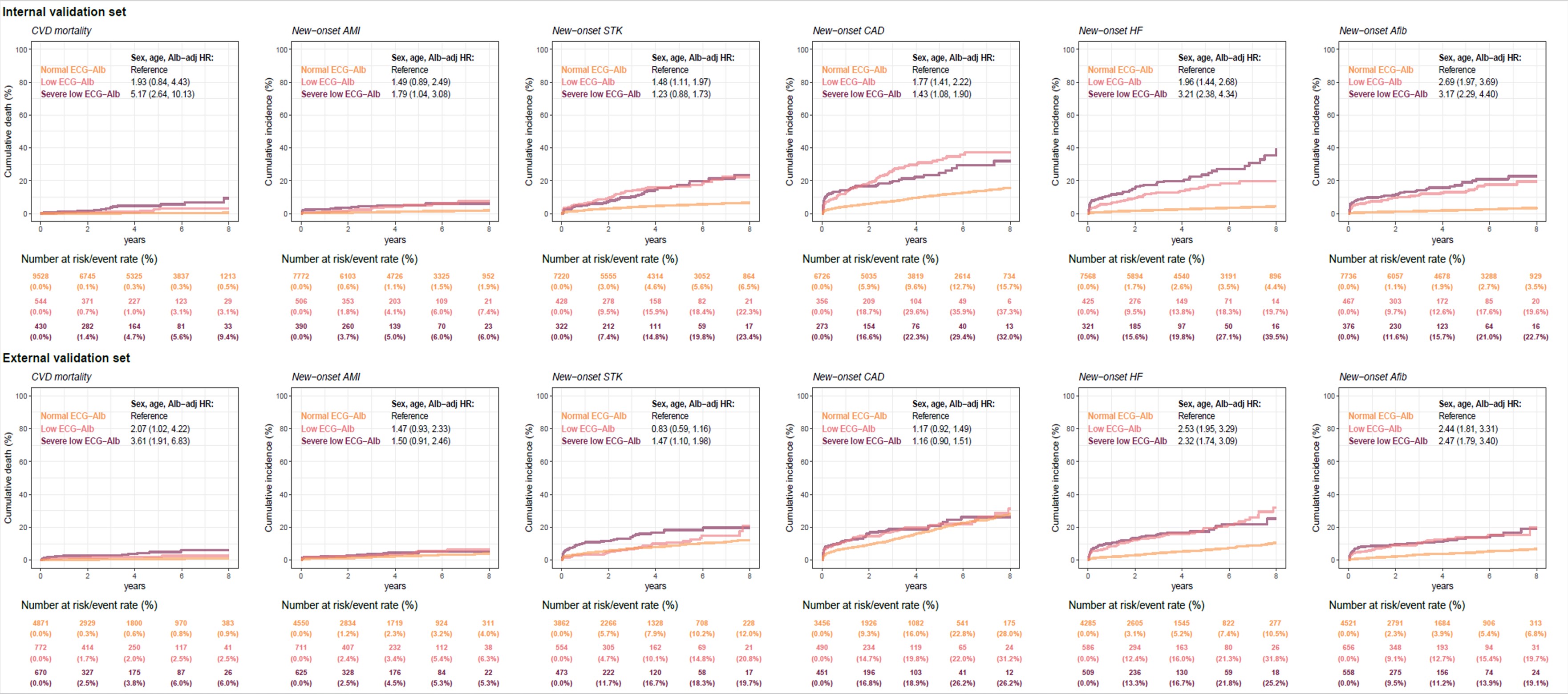

Supplement: Supplementary file 2 [file Image_2.jpg]
